# Knowledge mapping of metformin use on cancers: a bibliometric analysis (2013–2023)

**DOI:** 10.3389/fphar.2024.1388253

**Published:** 2024-08-13

**Authors:** Chaomin Pan, Yiyi Wei, Jingping Dai, Li Yang, Zhuoyu Ding, Xinke Wang

**Affiliations:** Guangdong Provincial Key Laboratory of Gastroenterology, Department of Gastroenterology, Nanfang Hospital, Southern Medical University, Guangzhou, China

**Keywords:** bibliometrics, metformin, cancers, citespace, VOSviewer

## Abstract

There is substantial evidence from clinical and preclinical studies suggesting an association between metformin use and a reduced risk of cancer. However, the effects of metformin use on cancers have not yet been subjected to bibliometric analysis. The goal of this study was to explore the potential effects of metformin use on cancers and to conduct a comprehensive assessment of research hotspots related to the use of metformin on cancers. The results of the literature analysis were visualized using various tools such as Adobe Illustrator CC 2018, VOSviewer, CiteSpace, and the R package “bibliometric.” The average annual publications from 2013 to 2023 was 372. In terms of journals and co-cited journals, a total of 1,064 journals published 1958 papers, and *Oncotarget* published the highest number of papers (n = 153, 7.81%), while *Cancer Research* (Co-citation = 5,125) was the most frequently cited journal. A total of 25,665 authors participated in the research on metformin use on cancers. Metformin has demonstrated improved outcomes in various types of cancer, including breast cancer (BC), lung cancer (LC), colorectal cancer (CRC), prostate cancer (PC), and pancreatic cancer. This bibliometric analysis reviews the current literature on the clinical data on metformin use on cancers and describes the preclinical evidence illustrating the potential mechanisms of metformin use on various cancers directly or indirectly.

## Introduction

Metformin is an oral hypoglycemic medication that promotes fatty acid oxidation, nonoxidative metabolism, and increases peripheral glucose consumption (Piper et al., 2019). Numerous observational cohort studies have indicated an association between metformin and a lower risk of various cancers, even when the data are inconclusive (Mallik and Chowdhury, 2018). Over the past decade, metformin has garnered interest from physicians and researchers owing to its potential benefits as an independent anticancer medication and as an anti-diabetic drug for preventing and treating cancer in diabetic patients (Kamarudin et al., 2019). The strongest correlations have been found between metformin and liver, breast, pancreatic, colorectal, and endometrial cancers (EC), and there may even be a preventive effect on PC (Mallik and Chowdhury, 2018).

Metformin has demonstrated improved outcomes in various types of cancer, including BC, LC, CRC, PC, pancreatic cancer, and others (Tian et al., 2017). In a retrospective study of patients with non-small cell lung cancer, chemotherapy with metformin, demonstrated better outcomes in terms of both overall survival (OS) (20.0 months vs. 13.1 months, *p* = 0.007) and progression-free survival (PFS) (8.4 months vs. 4.7 months, *p* = 0.002) as compared to insulin (Tan et al., 2011). A meta-analysis conducted in 2015 revealed that BC patients taking metformin had a significantly lower all-cause mortality rate (Hazard Ratio (*HR*) 0.652, 95% confidence interval (*CI*) 0.488–0.873; *p* = 0.004) (Yang et al., 2015). Pre-diagnostic administration of metformin was associated with a decreased risk of CRC (Odds Ratio (*OR*) = 0.754, *95% CI* 0.623–0.912, *p* = 0.004) in two large-scale, population-based, case-control studies (Rennert et al., 2020). *In vitro* studies have shown that metformin therapy reduced viability and increased apoptosis of PC cells (Wang et al., 2015). A meta-analysis of a single experiment showed that metformin had an obvious impact on radiographic PFS (HR 0.48, 95% CI 0.34–0.70) (Joshua et al., 2022). Strong epidemiological evidence has established a connection between metformin and a reduced risk of pancreatic ductal adenocarcinoma. For instance, the metformin group (n = 117) had a 2-year survival rate of 30.1%, whereas the non-metformin group (n = 185) had a rate of 15.4% (*p* = 0.004, χ2 test). Furthermore, the metformin group showed a better OS compared to the non-metformin group (15.2 months vs 11.1 months, *p* = 0.004, log-rank test) (Sadeghi et al., 2012).

A bibliometric analysis examines the quantity and quality of publications within a specific area of study (Pei et al., 2022). Programs such as CiteSpace (Synnestvedt et al., 2005), VOSviewer (Yeung et al., 2020), R package “bibliometric” (Li et al., 2020), and Adobe Illustrator CC 2018 can be used to depict the results of the literature analysis. However, there have been no bibliometric studies on metformin use on cancers. The distribution of countries, publications, authors, keywords, references, co-occurrence, and co-citations related to the topic of metformin use on cancers were depicted in this study. This study aimed to identify the major contributing factors and delineate the current research status within this domain, thereby providing valuable insights for clinical professionals and researchers interested in this field.

## Materials and Methods

### Search approach

**FIGURE 1:**
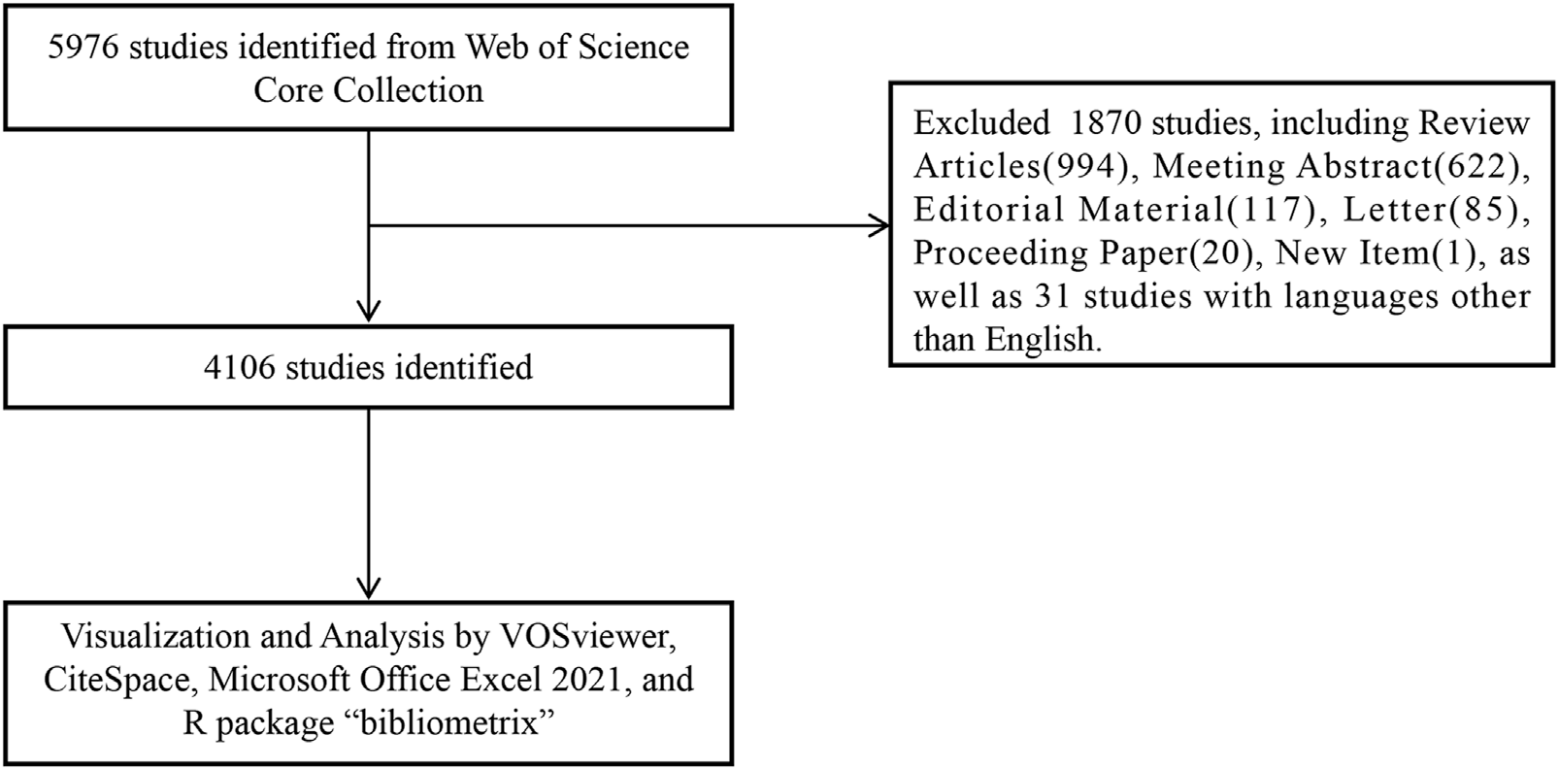
The procedure for data retrieval and collection.

We conducted a search for publications on 2 September 2023, using the Web of Science Core Collection (WoSCC) database (https://www.webofscience.com/wos/woscc/basic-search). The research formula used was ((TS = (*Metformin* OR *Glucophage* OR *N, N-1 dimethylbiguanide* OR *N-1,1-dimethylbiguanide*) AND TS = (*Cancers* OR *Neoplasms* OR *Tumors* OR *Carcinomas*)). The index data ranged from 1 January 2013, to 1 September 2023. The document type was specified as “articles” and the language was set to “English” (Figure 1). The manuscript containing the review articles would duplicate the calculated data from the manuscript containing the articles. Considering the search of manuscripts from 2013 to 2023, we excluded review papers from the analysis, as the literature cited in the review articles may have been published beyond these prescribed times.

### Graphical representation

VOSviewer (version 1.6.19) (van Eck and Waltman, 2010) is a widely used tool for the construction and visualization of bibliometric maps, enabling the analysis of collaboration, co-citation, and co-occurrence networks. In this study, VOSviewer software was used to conduct key analyses, including country and institution analysis, journal and co-cited journal analysis, author and co-cited author analysis, and keyword co-occurrence analysis (Chen et al., 2023). Each node within the VOSviewer maps represents a distinct entity, such as a country, journal, institution, or author. The size of the nodes indicated the quantity and type of publications, occurrences, and citations associated with them. Color indicated the classification of country, institution, journal, and author. Additionally, the strength of collaboration co-citation between items was represented by the color and thickness of the lines connecting the nodes.

CiteSpace (version 6.2. R4) is another valuable citation visualization analysis tool developed by Professor Chen C. Its primary purpose was to facilitate the identification of prospective data within scientific publications (Synnestvedt et al., 2005). Using visualization tools, it was possible to demonstrate how knowledge was gathered, distributed, and managed (Chen, 2004). In our investigation, we created a journal dual-map overlay using CiteSpace and performed a Citation Burst keyword analysis.

To conduct a thematic evolution analysis and establish a global distribution network of articles about metformin use on cancers, the R package “bibliometric” (version 4.2.3) (https://www.bibliometrix.org) was utilized. The quartile and impact factor of the journals were obtained from the Journal Citation Reports 2022 (https://jcr.clarivate.com/jcr/browse-journals). Furthermore, Microsoft Office Excel 2021 was used for the quantitative analysis.

## Results

### Global trend in publication output

**FIGURE 2:**
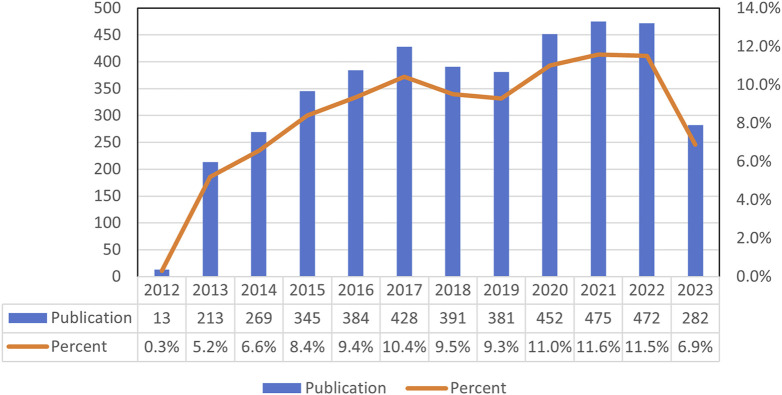
Global publishing production of metformin use on cancers.

A total of 4,106 studies on the effects of metformin use on cancers were conducted between 2013 and 2023. Figure 2 illustrated the global trend in publications and total citations for metformin use on cancers. Based on the annual growth rate of publications, 2021 showed the highest rate of publication growth, at 11.6%. Except for 2019, the number of metformin-related papers on cancers had steadily increased. In 2013, there was a significant increase of 5.2% in metformin papers in the field of cancer, representing a 4.9% increase from the previous year (Figure 2). In general, over the past decade, there has been a notable increase in metformin-related papers, indicating sustained research focus in this area.

#### Distribution of country, region, and institution

**FIGURE 3:**
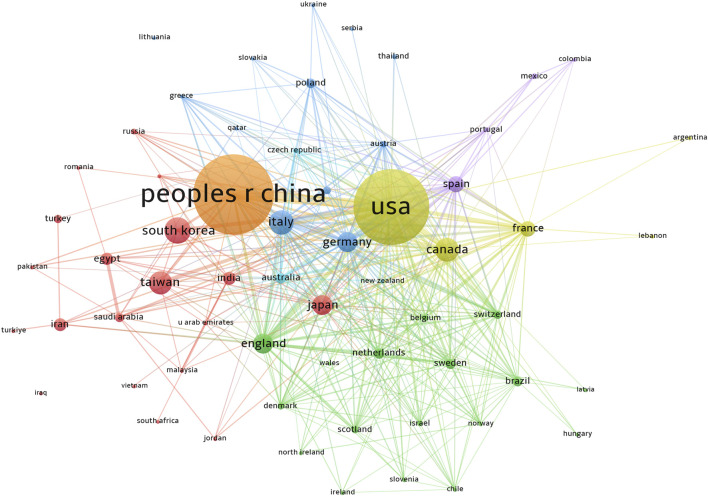
Country and region publications related to metformin use on cancers. VOSviewer was used to create a visualization map of the countries’ cooperative network. Colors represented different countries and regions. The sizes of the nodes were weighted by citations. People r china, The People’s Republic of China; United States, United States of America; south korea, South Korea; italy, Italy; canada, Canada; taiwan, Taiwan; japan, Japan; england, England; germany, Germany; spain, Spain; france, France.

Publications related to this topic originated from 61 countries and regions, and 4,636 institutions. The top ten countries and regions spread across Asia, North America, and Europe, with the highest number of publications in Europe, followed by Asia (Table 1). Among these countries and regions, China (n = 1,239, 22.56%) had the highest number of publications, followed by the United States of America (United States) (n = 1,143, 20.81%), South Korea (n = 254, 4.62%), and Italy (n = 232, 4.22%). Combined publications from China and the United States accounted for almost half of the total publications (43.37%). Notably, there was extensive cooperation between the countries. For instance, China had intensive collaborative partnerships with South Korea, Japan, the United States, Italy, Germany, Canada, and England (Figure 3).

**TABLE 1:** Top ten most productive countries, regions, and institutions on research of metformin use on cancers.

Rank	Country	Counts	Institution	Counts
1	China (Asia)	1,239 (22.56%)	Shanghai Jiao Tong University (China)	96 (1.48%)
2	the United States (North America)	1,143 (20.81%)	National Taiwan University Hospital (China)	76 (1.17%)
3	South Korea (Asia)	254 (4.62%)	University Texas MD Anderson Cancer Center (the United States)	75 (1.16%)
4	Italy (Europe)	232 (4.22%)	McGill University (Canada)	72 (1.11%)
5	Canada (North America)	219 (3.99%)	National Taiwan University (China)	72 (1.11%)
6	Japan (Asia)	175 (3.19%)	Fudan University (China)	66 (1.02%)
7	England (Europe)	174 (3.17%)	National Health Research Institutes (China)	61 (0.94%)
8	Germany (Europe)	170 (3.09%)	Zhejiang University (China)	57 (0.88%)
9	Spain (Europe)	129 (2.35%)	Shandong University (China)	54 (0.83%)
10	France (Europe)	123 (2.24%)	Mayo clinic (the United States)	52 (0.80%)

**FIGURE 4:**
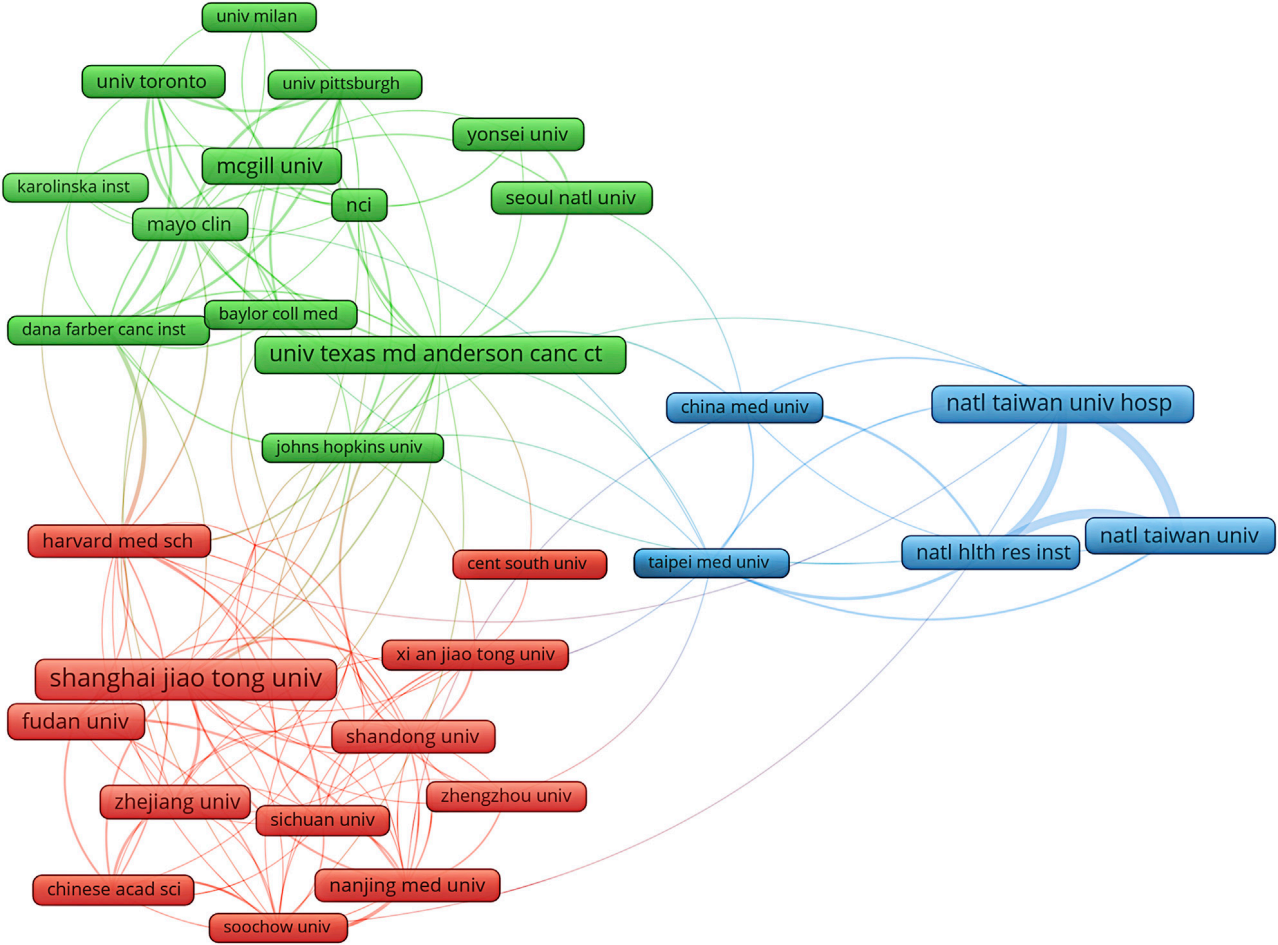
The diagram of institutions’ collaboration network in research on metformin use on cancers. The different colors represented different clusters, which were sorted according to the strength of collaboration. Shanghai jiao tong univ, Shanghai Jiao Tong University; natl taiwan univ hosp, National Taiwan University Hospital; univ texas md anderson canc ctr, University Texas MD Anderson Cancer Center; mcgill univ, McGill University; natl taiwan univ, National Taiwan University; fudan univ, Fudan University; natl hlth res inst, National Health Research Institutes; zhejiang univ, Zhejiang University; shandong univ, Shandong University; mayo clin, Mayo clinic.

Based on publication counts, Table 1 included the top ten most productive institutions, with institutions from China and the United States prominently represented in scientific research. These top ten institutions were in three countries, with the majority (7/10) situated in China. The four institutions that published the most relevant papers were National Taiwan University Hospital (n = 76, 1.17%), University of Texas MD Anderson Cancer Center (n = 75, 1.16%), McGill University (n = 71, 1.11%), and National Taiwan University (n = 72, 1.11%) (Table 1). Based on the quantity and correlation of publications from each institution, we selected 33 institutions (Figure 4). The close collaboration and active cooperation among Shanghai Jiao Tong University, Fudan University, Shandong University, and Zhejiang University were depicted in Figure 4.

## Distributions of journals and co-cited journals

A total of 1,064 journals published 1958 documents related to metformin use on cancers. The highest number of papers was published in *Oncotarget* (n = 153, 7.81%), followed by *Plos One* (n = 123, 6.28%), *Scientific Reports* (n = 110, 5.62%), and *Cancer* (n = 60, 3.06%) (Table 2). Among the top ten journals, the *International Journal of Cancer* had the highest impact factor (IF = 6.40), followed by *Frontiers in Pharmacology* (IF = 5.60) and the *International Journal of Molecular Sciences* (IF = 5.60).

**TABLE 2:** Top ten journals and co-cited journals related to the research of metformin use on cancers.

Rank	Journal	Count	If	Q	Co-cited journal	Co-citation	If
1	Oncotarget ^a^	153 (7.81%)	5.17	Q1	Cancer Research	5,125	11.20
2	Plos One	123 (6.28%)	3.70	Q2	Plos One	4,055	3.70
3	Scientific Reports	110 (5.62%)	4.60	Q2	Diabetes Care	3,513	16.20
4	Cancers	60 (3.06%)	5.20	Q2	Oncotarget	3,151	5.17
5	International Journal of Molecular Sciences	59 (3.01%)	5.60	Q2	Nature	2,745	64.80
6	BMC Cancer	50 (2.55%)	3.80	Q2	Journal of Biological Chemistry	2,717	4.80
7	Frontiers in Oncology	44 (2.25%)	4.70	Q2	Proceedings of the National Academy of Sciences of the United States of America	2,502	11.10
8	Oncology Letters	44 (2.25%)	2.90	Q3	Journal of Clinical Oncology	2,487	45.40
9	Frontiers in Pharmacology	36 (1.84%)	5.60	Q1	Clinical Cancer Research	2,297	11.50
10	International Journal of Cancer	35 (1.79%)	6.40	Q1	Cell	2,183	64.50

Oncotarget^a^: The impact factor for Oncotarget (5.17, Q1) was obtained from Journal Citation Reports 2016, while the others were obtained from Journal Citation Reports 2022.

**FIGURE 5:**
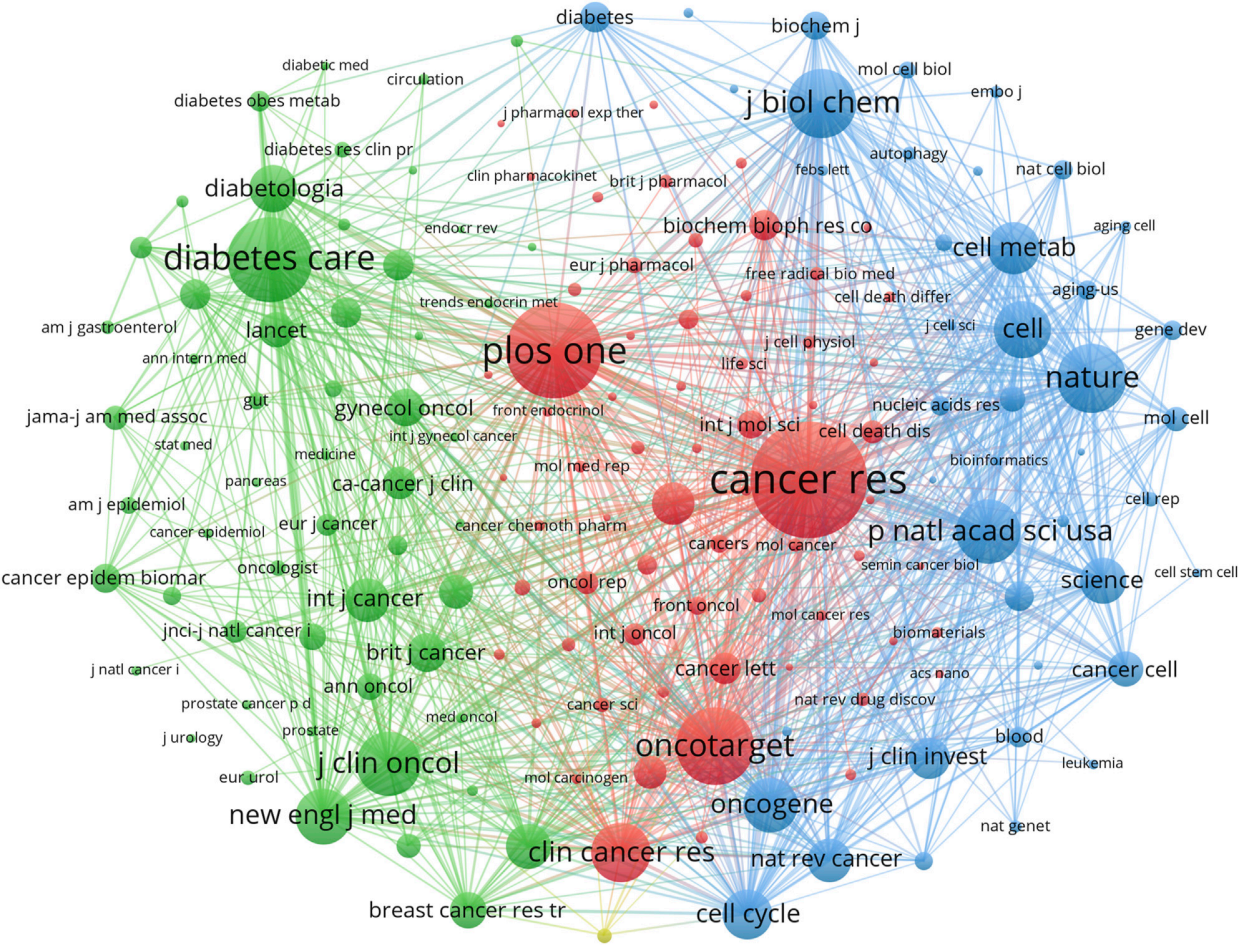
Co-cited journals on research of metformin use on cancers. Different colors represented different co-cited journals. The sides of the nodes were weighted by citations.

Co-cited journals are those that are often cited together by other researchers, and their influence was examined using co-citation analysis. Among the top ten co-cited journals, four were cited more than 3,000 times. The most cited journal was *Cancer Research* (Co-citation = 5,125), followed by *Plos One* (Co-citation = 4,055), *Diabetes Care* (Co-citation = 3,513), and *Oncotarget* (Co-citation = 3,151) (Table 2). Furthermore, *Nature* had the highest impact factor (IF = 64.80) in 2022, followed by *Cell* (IF = 64.50) in 2022. To create a co-citation network, journals with a minimum of 200 co-citations were filtered out (Figure 5). *Cancer Research* exhibited significant co-citation associations with *Plos One*, *Oncotarget*, *Clinical Cancer Research*, and others (Figure 5). These findings suggested that this field had advanced greatly thanks to journals such as *Cancer Research*.

**FIGURE 6:**
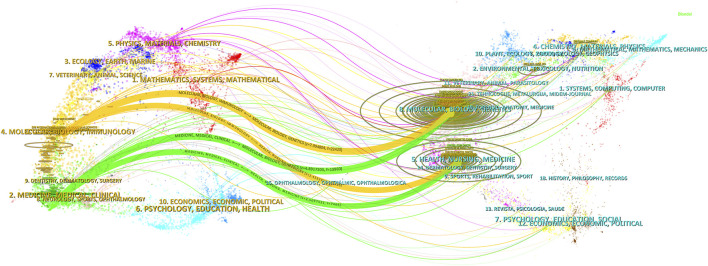
Dual-map overlay of journals pertaining to research of metformin use on cancers. The designations matched the wide range of study topics covered by the publications. There were different colored lines for the different reference paths, and the citing journals were on the left side, whereas the other side of the map represented the cited journals. The various courses of references, starting at the citing map and concluding at the cited map, were represented by distinct colored lines. The path widths were scaled according to the citation frequency on the z-score scale.

The distribution of journal topics was depicted by the dual-map overlay of journals in Figure 6, which displayed the citation links of journals and co-cited journals. The clusters of citing journals were presented on the left side of the map, while the clusters of cited journals were on the right side. Besides, Figure 6 showed that the flows from the citing subject categories to the cited subject categories were primarily represented by two orange pathways (from Molecular/Biology/Genetics and Health/Nursing/Medicine to Molecular/Biology/Immunology) and two green pathways (from Molecular/Biology/Genetics and Health/Nursing/Medicine to Medicine/Medical/Clinical).

A total of 25,665 authors participated in the research on metformin use on cancers. Among the top ten most creative and productive authors and co-cited authors, Tseng Chin-hsiao was the most prolific (Table 3). Tseng Chin-hsiao had published the most relevant works, followed by Menendez Javier A. and Li. Among the 73,764 authors, 115 received a minimum of 10 citations. Six authors were co-cited more than 400 times among the top ten co-cited authors (Table 3). The most co-cited author was Tseng Chin-hsiao (n = 955), followed by Ben Sahra I (n = 827), and Evans JM (n = 661).

**TABLE 3:** Top ten most creative authors and co-cited authors on research of metformin use on cancers.

Rank	Authors	Count	Co-cited authors	Citations
1	Tseng Chin-hsiao	55	Tseng Chin-hsiao	955
2	Menendez Javier A	27	Ben Sahra I	827
3	Li	21	Evans JM	661
4	Joven Jorge	19	Dowling Rjo	571
5	Zarghami Nosratollah	19	Hardie DG	494
6	Cuyas, Elisabet	18	Zakikhani M	474
7	Martin-castile Begona	16	Anisimov Vladimir N	379
8	Pollak Michael N	16	Hirsch HA	376
9	Zhang Wei	16	Currie CJ	365
10	Pollak Michael	15	Pollak Michael	361

### Co-cited references

In our research, there were 111,691 co-cited references about metformin use on cancers over the past decade. Each reference in the top ten co-cited references (Table 4) had at least 240 co-citations. Evans JM received the highest number of citations at 651.

**TABLE 4:** Top ten co-cited references on research of metformin use on cancers.

Rank	Co-cited References	Citations
1	Evans JM, 2005, Bmj-brit Med J, V330, P1304	651
2	Zhou GC, 2001, J Clin Invest, V108, P1167	335
3	Zakikhani M, 2006, Cancer Res, V66, P10269	331
4	Ben Sahra I, 2008, Oncogene, V27, P3576	315
5	Libby G, 2009, Diabetes Care, V32, P1620	309
6	Decensi A, 2010, Cancer Prev Res, V3, P1451	301
7	Jiralerspong S, 2009, J Clin Oncol, V27, P3297	269
8	Owen MR, 2000, Biochem J, V348, P607	264
9	Hirsch HA, 2009, Cancer Res, V69, P7507	255
10	Dowling Rjo, 2007, Cancer Res, V67, P10804	240

Keywords with citation bursts.

## Keywords with citation bursts

**FIGURE 7:**
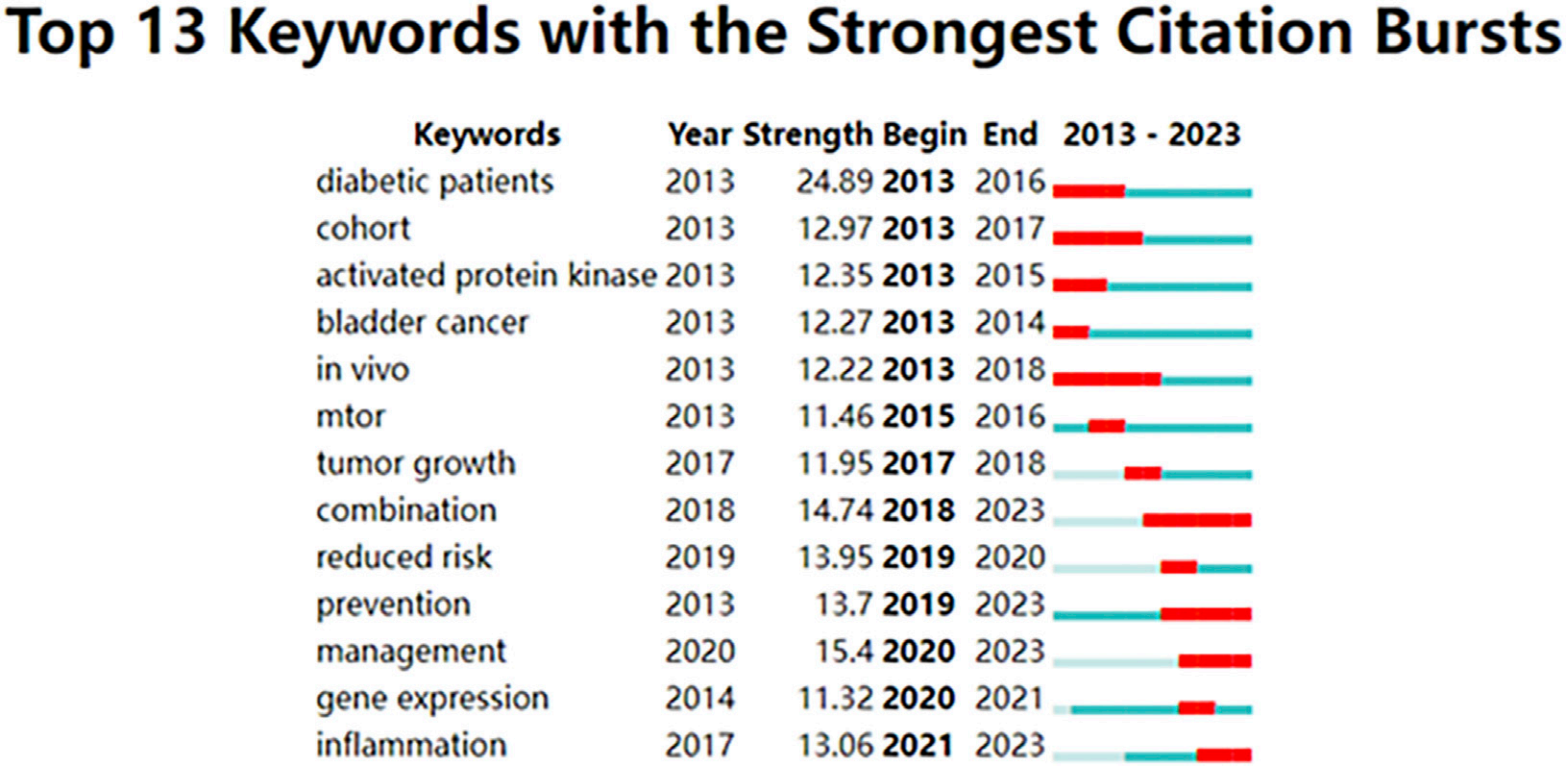
Top 13 keywords with robust citation bursts. High citations in that year were shown by a red bar.

Keywords with citation bursts were those that received a significant number of citations over time from scholars working in related fields. In our analysis, CiteSpace identified 13 keywords with substantial citation bursts (Figure 7). Figure 7 represented these bursts using bars corresponding to years, with the red bar indicating appearances as early as 2013 and as late as 2023. “Diabetic patients” was the term with the strongest citation burst (Strength = 24.89), followed by “management” (Strength = 15.4). These 13 keywords had a burst strength range of 11.32 to 24.89, and an endurance strength of two to six years.

## Hotspots and frontiers

Through co-occurrence analysis of specific keywords, we could rapidly identify future hotspot directions within a particular field. Table 5 displayed the top 20 high-frequency keywords extracted from abstracts and titles that met the established criteria for metformin use on cancers. The prominence of keywords such as metformin, BC, diabetes, and adenosine monophosphate-activated protein kinase (AMPK), appearing more than 200 times, reflects the primary research direction of metformin use on cancers.

**TABLE 5:** Top 20 keywords related to the research of metformin in cancers.

Rank	Keywords	Counts	Rank	Keywords	Counts
1	metformin	1802	11	hepatocellular carcinoma	89
2	breast cancer	240	12	type 2 diabetes	88
3	diabetes	225	13	endometrial cancer	85
4	ampk	201	14	metabolism	85
5	apoptosis	187	15	pancreatic cancer	83
6	diabetes mellitus	187	16	ovarian cancer	80
7	cancer	162	17	survival	79
8	colorectal cancer	115	18	mtor	74
9	prostate cancer	111	19	lung cancer	72
10	autophagy	96	20	insulin	68

**FIGURE 8:**
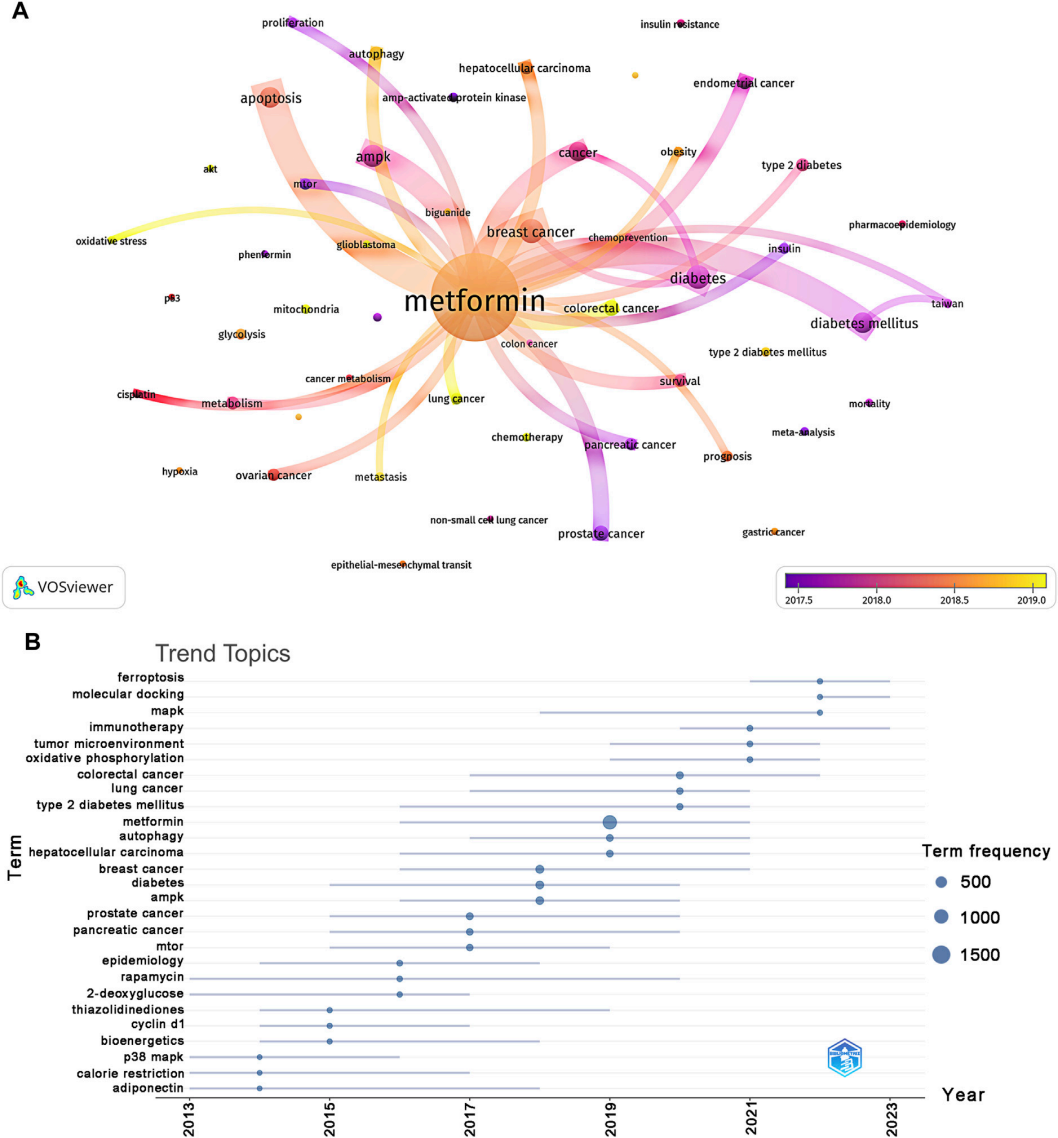
Keyword cluster analysis **(A)** and analysis of trending topics **(B)**. The color of the nodes corresponded to the average year in which the keyword appeared. The sides of the nodes represented the counts of keywords.

Using VOSviewer, we filtered out keywords that occurred 30 times or more, as shown in Figure 8A. The thickness of the connecting lines between nodes represented the strength of the relationship between keywords. We identified five distinct clusters, each representing a unique area of the study direction. The yellow cluster consisted of 21 keywords, including AKT, AMPK, biguanide, cancer metabolism, cancer stem cells, chemoresistance, chemotherapy, cisplatin, colon cancer, glioblastoma, glycolysis, hypoxia, LC, metabolism, metformin, mitochondria, ovarian cancer, oxidative stress, p53, and phenformin. According to trend topic analysis, metformin, diabetes, BC, and AMPK were the most frequently used keywords between 2013 and 2023 (Figure 8B). Furthermore, rapamycin and thiazolidinediones have emerged as the primary subjects of investigation. Since 2021, researchers have actively explored the pathogenesis and the therapeutic potential of metformin use on cancers, with oxidative phosphorylation, tumor microenvironment, and immunotherapy emerging as key areas of focus. Additionally, the keywords mitogen-activated protein kinase (MAPK), molecular docking, and ferroptosis have been frequently used over the past 2 years (2022–2023), indicating their representation in the current research hotspots for metformin use on cancers.

### Clinical trials of metformin use on various cancers

**FIGURE 9:**
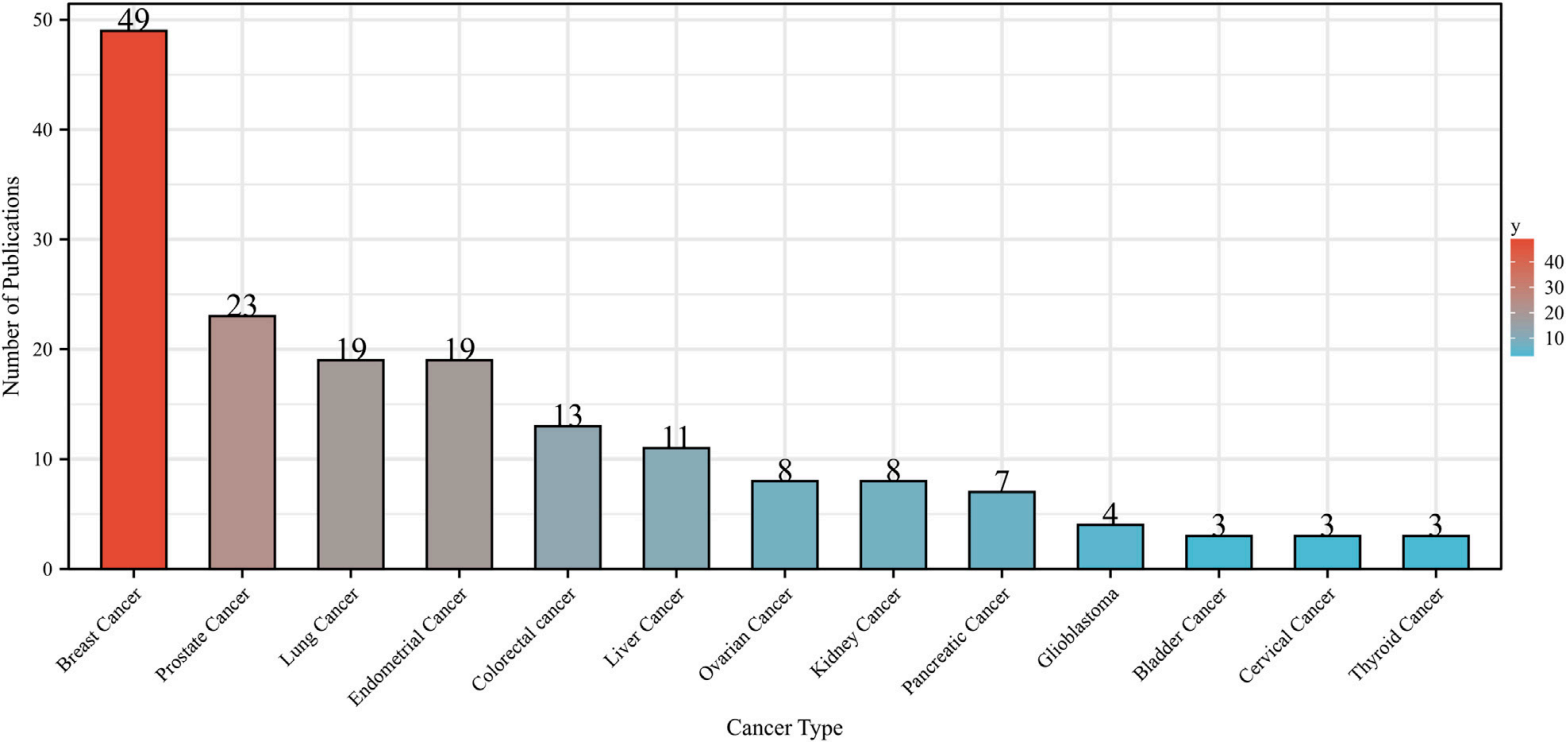
Number of publications about metformin use on various cancers in clinical trials from 2013 to 2023. Different colors represented different types of cancer.

Metformin was a widely used medication to treat diabetes and may have some anticancer effects (Dankner et al., 2019). We investigated the literature from 2013 to 2023, focusing only on clinical studies, to evaluate the potential impact of metformin use on various cancers. Figure 9 only displayed the top 13 tumors with the most papers about metformin use on cancers in clinical trials. BC accounted for the majority of the papers, followed by PC, LC, and EC. This was because metformin has been shown to improve metabolic variables, such as insulin, glucose, leptin, and highly sensitive C-reactive protein (Goodwin et al., 2015). The use of metformin on cancers related to bladder, cervical, and thyroid tumors was the subject of only three articles. This implied that more research about metformin use on various cancer domains was still pending.

## Discussion

### General information

Between 2013 and 2023, there was a significant increase in the number of clinical or preclinical publications focusing on metformin use on cancers. Clinical studies, also known as clinical trials, are a critical part of medical research. The types of clinical studies included meta-analyses, case reports, cross-sectional studies, case-control studies, cohort studies, and randomized controlled trials (RCT). Preclinical research is a critical step in translational research that provides information on the potential of medical devices, therapeutic drugs, or medical treatments before being tested in humans (Wu et al., 2023). During this period, the average number of preclinical and clinical publications each year was 372, indicating a burgeoning trend in research pertaining to the role of metformin use on cancers.

When it comes to journals, most of the research about metformin use on cancers has been published in *Oncotarget* (IF = 5.17, Q1), making it the most widely read journal in this field. However, the impact factor of *Oncotarget* was stuck in 2016, and the Science Citation Index no longer included articles from *Oncotarget* in 2018. The *International Journal of Cancer* (IF = 6.40, Q1) had the greatest impact among other journals, followed by *Frontiers in Pharmacology* (IF = 5.60, Q1) and *International Journal of Molecular Sciences* (IF = 5.60, Q2). Regarding co-cited journals, we discovered that most were Q2 journals. Undoubtedly, these journals were of high quality, encouraging further investigation into the study of metformin use on cancers. Moreover, the current research about metformin use on cancers has been primarily published in journals related to Molecular Biology and Immunology, while few studies are published in clinically related journals, suggesting that most of the research is still in the early stages.

From the perspective of authors alongside co-cited authors, both Tseng Chin-hsiao and Pollak Michael were in the top ten list. Tseng Chin-hsiao authored the largest number of articles, accounting for 55 publications between 2013 and 2023. He pointed out that metformin use may reduce the risk of cancer in Taiwanese patients with type 2 diabetes mellitus (T2DM), including bladder cancer (Tseng, 2014a), malignant brain tumors (Tseng, 2021), esophageal cancer (Tseng, 2017a), skin cancer (Tseng, 2018), endometrial cancer risk (Tseng, 2015), lung cancer (Tseng, 2017b), cervical cancer (Tseng, 2016a), oral cancer (Tseng, 2016b), and thyroid cancer (Tseng, 2014b) based on retrospective cohort studies. Menendez Javier A, an expert on the regulatory mechanism of metformin use in BC treatment in many preclinical studies, was the second most published author. His findings demonstrated that metformin inhibited the overexpression of HER2 (erbB-2) oncoprotein in human BC cells by blocking the mammalian target of rapamycin complex 1 (mTORC1) effector p70S6K1 (Vazquez-Martin et al., 2009). Additionally, the metastasis-associated protein CD24 was reduced in triple-negative BC cells by metformin (Oliveras-Ferraros et al., 2011). The capacity of metformin to alter the expression of miRNAs may also be related to its effect on the development of BC (Oliveras-Ferraros et al., 2011), such as the suppression of miRNA let-7a and the promotion of oncomiR miRNA-181a.

Pollak Michael, with 15 publications and 361 citations to his name, concluded the impact of metformin on the microbiota and immune system could have implications not only for diabetes treatment but also for other suggested indications, such as oncology and aging (Pollak, 2017). Pollak Michael also revealed metformin inhibited the mTORC1 signaling pathway in a preclinical study, which was often overexpressed in hepatocellular carcinoma (Bhat et al., 2017). In addition, his research team has been engaged in the development of more efficacious medication combinations and in elucidating the potential mechanisms underlying the heterogeneous response of cancer cells to metformin. He proposed that effective anticancer medicines could be achieved by focusing on the modulation of mitochondrial function and the biosynthesis of nicotinamide adenine dinucleotide (Parisotto et al., 2022). Upon validation of metformin’s therapeutic potential in cancers, he and his team members investigated the mechanism underlying the role of metformin use on cancers at clinical dosages. Through their endeavors, they discovered that metformin increased 18-fluorodeoxyglucose (18-FDG) uptake in tumors, activated various mitochondrial metabolic pathways, and lowered the levels of mitochondrial metabolites. Based on these findings, they concluded that the anti-tumor effect of metformin use on primary BC treatment may be characterized by the mitochondrial response to the drug (Lord et al., 2018).

### Knowledge base

A co-cited reference is one that has been cited in multiple publications. These co-cited references play a significant role in shaping future research in a specific field (Wu et al., 2022). Our bibliometric analysis identified the top ten co-cited references with the highest number of co-citations. Among these, a pilot case-control study by Evans et al., conducted in 2005, was the most frequently referenced. This study revealed that metformin treatment in patients with T2DM could potentially reduce their risk of developing cancer, as demonstrated by the use of established techniques (Evans et al., 2005).

Another co-citation reference by Zhou highlighted that metformin-stimulated activation of AMPK resulted in decreased glucose synthesis in the liver of metformin-treated rats (Zhou et al., 2001). Additionally, Zakikhani discovered that metformin could inhibit the growth of BC cells through AMPK in a preclinical study (Zakikhani et al., 2006). Further research by Ben indicated that metformin exhibited anti-tumoral effects both *in vitro* and *in vivo* by reducing the level of cyclin D1 (Ben Sahra et al., 2008). Libby’s study demonstrated the mechanism of action of metformin involves activating AMP-activated protein kinase, leading to the suppression of tumor formation, inhibition of cell proliferation, and reduced risk of cancer in individuals with T2DM (0.63, 0.53–0.75) in a preclinical study (Libby et al., 2009).

To assess the impact of metformin use on cancer incidence and mortality in patients with diabetes, Bonanni conducted a literature review and meta-analysis. The findings showed that individuals taking metformin had an overall 31% reduction in the relative risk (Risk Ratio (*RR*) = 0.69, *95% CI* 0.61–0.79) compared to those using other antidiabetic medications (Decensi et al., 2010). Jiralerspong reported diabetic patients with BC who received metformin and neoadjuvant chemotherapy had a higher rate of pathological complete response compared to those without metformin administration in a retrospective cohort study (Jiralerspong et al., 2009). Owen presented data demonstrating that metformin inhibited complex one of the mitochondrial respiratory chain, thereby exerting its anti-diabetic effects in a preclinical study (Owen et al., 2000). Hisch observed that low doses of metformin, a commonly used diabetes medication, impeded cellular transformation and eliminated cancer stem cells in different forms of BC in a preclinical study (Hirsch et al., 2009). Furthermore, Dowling highlighted that metformin-mediated AMPK activation led to mechanistic target of rapamycin (mTOR) inhibition and a decrease in translation initiation, suggesting a potential mechanism of action for the drug in slowing down the proliferation of cancer cells in a preclinical study (Dowling et al., 2007).

## Hotspots and frontiers

Keywords with citation bursts indicated new ideas within a specific field of study, as evidenced by their numerous citations in recent years. As shown in Table 5, the primary focus of research on keywords with high citation bursts suggested ongoing investigations into the biological function and mechanism of metformin use on cancers, and its potential association with various types of cancer. By conducting trend topic analysis and examining keywords with citation bursts, the distribution and the evolution of hotspots in metformin-related cancer research, such as CRC, oxidative phosphorylation, tumor microenvironment, immunotherapy, AMPK, molecular docking, and ferroptosis, could be quickly identified.

### The molecular mechanism of metformin use on cancers in preclinical studies

**FIGURE 10:**
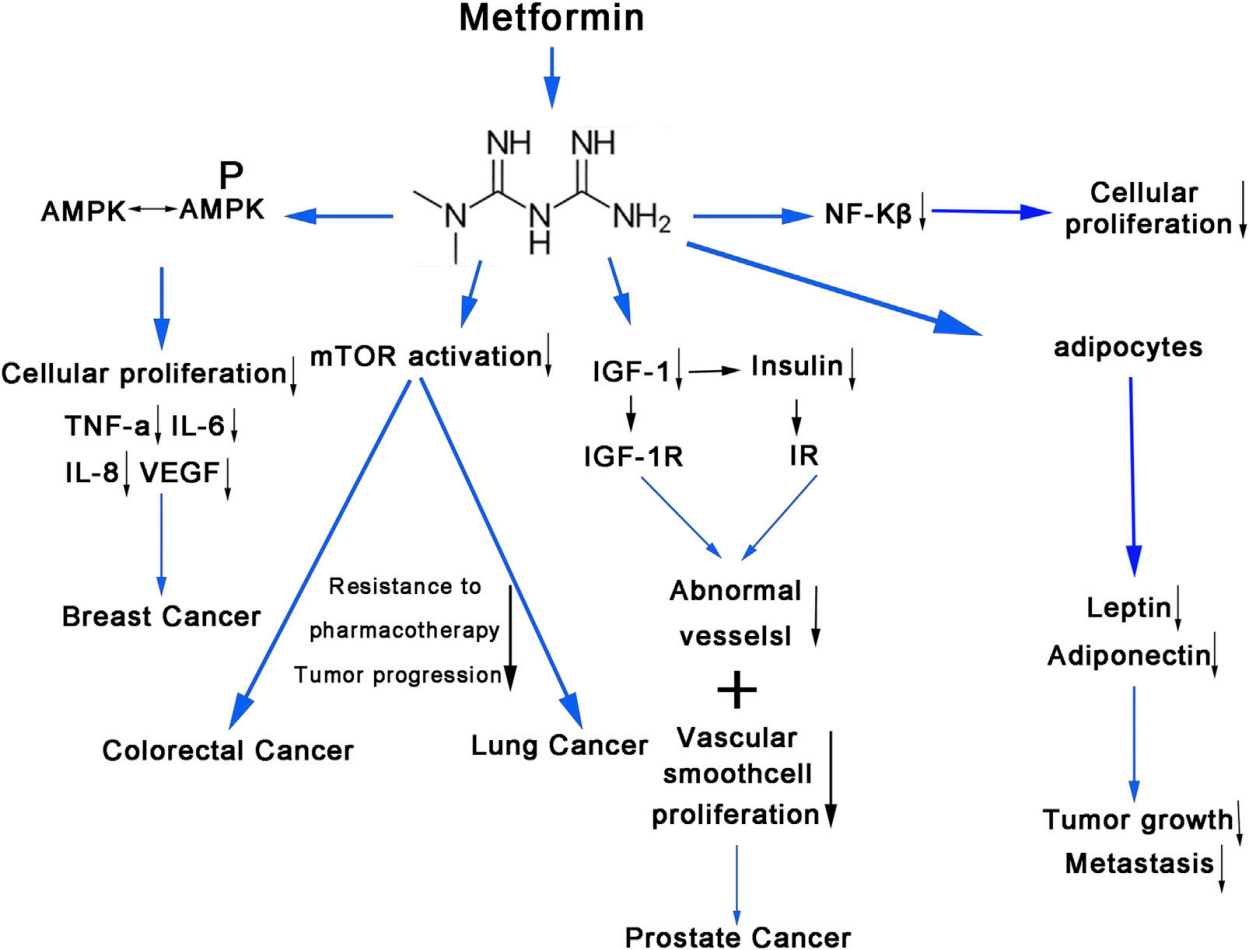
The molecular mechanism of metformin use on cancers. The anti-hyperglycemic effects of metformin affect cancer cell metabolism through various signaling pathways. AMPK, adenosine monophosphate-activated protein kinase; TNF-α, tumor necrosis factor alpha; IL-6, inteleukin-6; IL-8, inteleukin-8; VEGF, vascular endothelial growth factor; mTOR, mammalian target of rapamycin; NF-kβ, nuclear factor kappa; IGF-1, insulin-like growth factor-1; IGF-1R, insulin-like growth factor-1 receptor; IR, insulin receptors.

Metformin’s anti-hyperglycemic actions appeared to be regulated through the activation of AMPK. Activation of AMPK has been associated with numerous significant effects on cancer cell metabolism, including the suppression of cellular proliferation (Faubert et al., 2015) and the reduction of pro-inflammatory cytokines such as tumor necrosis factor (TNF)-α, interleukin (IL)-6, IL-8, and vascular endothelial growth factor (VEGF) (Takemura et al., 2007). Metformin has demonstrated its ability to inhibit mTOR activation in several cancer types, including thyroid, breast, lung, and leukemia. Overactivation of mTOR in cancer was associated with tumor growth, drug resistance, and a poorer prognosis (Hay and Sonenberg, 2004). Metformin was also an insulin sensitizer, leading to lower levels of insulin-binding proteins and plasma insulin, subsequently resulting in decreased levels of insulin-like growth factor-1 (IGF-1). Insulin and IGF-1 have been found to promote the development of abnormal blood vessels and proliferation of vascular smooth muscle cells, which contribute to neoplastic processes and metastasis of breast, colorectal, and prostate cancers (Key et al., 2010). Han et al. established a connection between elevated serum leptin levels and tumor growth and metastasis (Han et al., 2005). Conversely, adiponectin appeared to exert an inhibitory effect on cancer development and possess anti-proliferative properties in cancer cells (Dos Santos et al., 2008). The anti-cancer effect of metformin may be attributed to its stimulation of adiponectin production and inhibition of sleeper cell generation (Dos Santos et al., 2008). Reduction in nuclear factor kappa B (NF-κβ) levels could result in decreased cancer cell proliferation and increased sensitivity to chemotherapeutic treatments. Metformin seemed to decrease the expression of NF-κβ, resulting in decreased cellular proliferation (Wang et al., 1999) (Figure 10).

### The clinical trials of metformin use on cancers

As illustrated in Figure 9, BC accounted for the majority of studies on the effect of metformin use on cancers. In patients with primary HER2-positive and hormone receptor-positive BC, patients with diabetes who did not receive metformin treatment had lower rates of disease-free survival (DFS) (multivariable *HR*, 1.40; *95% CI*, 1.01–1.94; *p* = 0.043) and OS (multivariable *HR*, 1.87; *95% CI*, 1.23–2.85; *p* = 0.004) than patients with diabetes who had received metformin treatment (Sonnenblick et al., 2017). In a randomized BC study, the difference between the metformin and placebo groups resulted in a statistically significant reduction in cancer antigen 15–3 (CA 15–3), a tumor marker and regulator of cellular metabolism (absolute geometric mean reduction in CA 15–3 = 7.7% vs 2.0%, *p* < 0.001) (Goodwin et al., 2021). Barakat et al. indicated a trend towards clinical complete response and pathological complete response was associated with higher serum metformin concentrations in locally advanced BC patients in an open-label RCT (Barakat et al., 2022). BC patients who experienced recurrence had higher levels of estrogens than those who did not recur (22.7 vs., 10.8 pg/mL; *p* = 0.05) (Rock et al., 2008). Metformin reduced estradiol levels in a phase III trial of non-diabetic BC participants who were randomly randomized to receive placebo or metformin, indicating a novel metformin effect that may be relevant to estrogen-sensitive cancers (Pimentel et al., 2021).

In PC, metformin treatment was safe for people without diabetes and produced objective responses to prostate-specific antigens. It may also induce disease stabilization in a multicenter phase 2 trial (Rothermundt et al., 2014). Furthermore, a phase II RCT in PC found that metformin added to radiation and androgen deprivation therapy was typically safe and well-tolerated, with no increase in rates of grade 2 gastrointestinal or genitourinary damage (Kim et al., 2021). In a randomized phase II study, the addition of metformin significantly reduced the risk of progression (*HR* = 0.31; *95% CI* = 0.12–0.78, *p* = 0.013) and death (*HR* = 0.42; *95% CI* = 0.18–0.94, *p* = 0.035) in non-small cell lung cancer and squamous cell carcinoma with high fluorodeoxyglucose uptake (Lee et al., 2021). During medroxyprogesterone acetate therapy, metformin prevented disease relapse in a phase II study of EC (Mitsuhashi et al., 2016). Furthermore, in an RCT, the group receiving metformin plus megestrol acetate had a greater 16-week treatment complete response rate (34.3 *versus* 20.7%, *OR* 2.0, *95% CI* 0.89–4.51, *p* = 0.09) for EC than the group receiving megestrol acetate alone (Yang et al., 2020).

After taking into account the aforementioned clinical data, we concluded that metformin use may be relevant to longer OS and DFS rates, more clinical complete response and pathological complete response, and reduced progression and death risk in various cancers. However, we could not determine whether metformin could be used alone in cancer treatment. Further studies on the effects of metformin on cancer are required.

### Advantage and shortcomings

This study offered notable advantages. First, it presented a comprehensive and systematic bibliometric analysis of metformin research on cancers for the first time, providing researchers with a thorough understanding of relevant studies. Second, our data analysis approach was highly objective as we employed three widely used bibliometric tools simultaneously, including VOSviewer and CiteSpace. Lastly, bibliometric analysis enabled us to gain a more comprehensive insight into the frontiers and hotspots of research compared to standard reviews.

However, this study had some limitations. First, we relied solely on data from the WoSCC database, thereby excluding valuable information from other databases and potentially overlooking important studies. Second, we only included articles and English-language research, which may result in incomplete literature collection and an underrepresentation of non-English writing samples. Third, due to insufficient data availability, publications from more than a decade ago were not fully integrated into our analysis. Furthermore, there is a lag in co-cited reference analyses, with newly published articles taking some time to accumulate sufficient citations. Lastly, due to space constraints, we only listed a few important mechanisms by which metformin inhibits cancers.

## Data Availability

The original contributions presented in the study are included in the article/supplementary material, further inquiries can be directed to the corresponding authors.
